# Advanced new strategies for metastatic cancer treatment by therapeutic stem cells and oncolytic virotherapy

**DOI:** 10.18632/oncotarget.11017

**Published:** 2016-08-02

**Authors:** Geon-Tae Park, Kyung-Chul Choi

**Affiliations:** ^1^ Laboratory of Biochemistry and Immunology, College of Veterinary Medicine, Chungbuk National University, Cheongju, Chungbuk, Republic of Korea; ^2^ TheraCell Bio & Science, Cheongju, Chungbuk, Republic of Korea

**Keywords:** metastatic cancer, therapeutic stem cell, oncolytic virotherapy, cancer treatment

## Abstract

The field of therapeutic stem cell and oncolytic virotherapy for cancer treatment has rapidly expanded over the past decade. Oncolytic viruses constitute a promising new class of anticancer agent because of their ability to selectively infect and destroy tumor cells. Engineering of viruses to express anticancer genes and specific cancer targeting molecules has led to the use of these systems as a novel platform of metastatic cancer therapy. In addition, stem cells have a cancer specific migratory capacity, which is available for metastatic cancer targeting. Prodrug activating enzyme or anticancer cytokine expressing stem cells successfully inhibited the proliferation of cancer cells. Preclinical models have clearly demonstrated anticancer activity of these two platforms against a number of different cancer types and metastatic cancer. Several systems using therapeutic stem cells or oncolytic virus have entered clinical trials, and promising results have led to late stage clinical development. Consequently, metastatic cancer therapies using stem cells and oncolytic viruses are extremely promising. The following review will focus on the metastatic cancer targeting mechanism of therapeutic stem cells and oncolytic viruses, and potential challenges ahead for advancing the field.

## INTRODUCTION

Cancer metastasis, which is a multiple process in which malignant cells spread from the primary site to colonize distant organs, is one of the greatest challenges in cancer treatment. Although metastasis is responsible for more than 90% of cancer associated mortality, it is difficult to diagnose and treat [1]. For many patients, metastasis has already occurred by the time when they are diagnosed with primary cancer. Depending on the type of cancer, metastasis shows various modalities. In breast cancer, metastasis of the primary tumor is difficult to detect and could remain latent for many years. Latent cells are then activated by unknown factors, resulting in formation of incurable lesions. Conversely, small cell lung cancer has often metastasized to multiple organs at the time of initial diagnosis [2]. Only a small number of patients with metastatic cancer can be successfully treated by conventional strategies such as surgical removal, chemotherapy and radiotherapy [3]. Therefore, it is necessary to develop a new strategy to prevent metastasis or treat existing metastases.

Conventional strategies of cancer therapy, including surgical resection, chemotherapy, radiotherapy and immunotherapy, have made significant contributions to cancer treatment. However, many people suffer from side-effects such as insufficient anti-cancer effects that involve drug resistance and systemic adverse reactions due to off target effects [4]. For example, one of advanced methods for metastatic breast cancer included monoclonal antibody that binds to the breast cancer specific HER2/neu receptor to interfere HER2 signaling pathway. As results, inhibition of downstream signaling pathways, cell cycle arrest and a reduction in angiogenesis occurred [5]. This method is very effective for metastatic breast cancer treatment, but it is only effective on breast cancer that expresses the HER2/neu receptor. In addition, some cancer patients acquired resistance to Trastuzumab during the period of administration [6, 7]. Another major problem associated with conventional cancer therapy is incomplete elimination of the invasive primary tumor masses, which cause metastasis to multiple organs, and tumor cell dormancy that leads to disease recurrence [8, 9]. Therefore, the recent goal of new therapies has been development of cancer treatments that have sufficient therapeutic capacity with little or no toxicity to normal cells. To achieve this goal, a better understanding of the detailed mechanisms of cancer using the latest biotechnology and innovative anti-cancer technologies is needed.

A mechanistic understanding of the metastatic process is important to development of anti-metastatic therapies that could reduce patient mortality. The metastatic process is initially derived from gene mutations that correlate with proliferative ability. Cells in normal tissues only divide when they receive growth stimulatory signals from other cells and stop dividing when they receive growth inhibitory signals; however, gene mutations providing the ability to be split ignore these signaling factors [10]. Additionally, these oncogenic mutations cause the cells to maintain progenitor like phenotypes and generate oncogenically transformed cells from normal cells. However, many studies using oncogene driven mouse models have shown that cancer did not automatically metastasize to distant organs and that oncogenic transformation is not sufficient for metastatic potential [11]. This is because metastasis is a very complicated process that involves a number of genes associated with tumor cell invasion from the primary tumor to the bloodstream, circulation and exit from the circulatory system to distant organs, and then angiogenesis and colonization of the distant organ [12]. Because of the complexity, metastatic cancer treatment appears to be difficult.

In this review, we will discuss recent strategies for the treatment of metastatic cancer based on stem cells and oncolytic viruses. Many stem cells have intrinsic tumor tropic properties that originate from chemokine interactions with cancer cells. Using this property, we can make a specific delivery system of anticancer molecules. Stem cells can migrate towards tumor microenvironments and eliminate tumors, enabling site specific delivery. Furthermore, stem cells can be modified to stably express various anticancer agents including cytokines and prodrug activating enzymes for induction of cancer apoptosis and removal of specific tumors. In addition, oncolytic viruses are a therapeutically useful system that can be used to selectively infect and damage tumor tissues without off target effects on normal tissues. Each virus has a specific cellular tropism that determines which tissues are preferentially infected. Viruses then increase in the tumors and destroy them, after which they infects another tumor cell. Viral oncotherapy can also be modified to increase tumor selectivity and enhance oncolytic activity. For example, some viruses have been modified to express capsid proteins that bind with specific cancer types and conditionally express the genes involved with the activation of host immune system. These two strategies will be able to complement the drawbacks of conventional cancer therapy.

## THERAPEUTIC STEM CELL FOR METASTATIC CANCER

### Specific tumor tropism of stem cells

Stem cells can trace cancer cells and tumor regions, which makes them very useful for tracing metastatic cancer and carrying anti-metastatic molecules. Various chemokine-chemokine receptor interactions are important to recognition of tumor cells and tumor tropism of stem cells. Stromal cell derived factor 1 alpha (SDF-1α) and its receptor, CSC chemokine receptor 4 (CXCR4), have been identified as key molecules responsible for the tropism of stem cells in many cancers [13]. According to the results, the SDF1α -CXCR4 signaling pathway plays a major role in the tumor specific migration of commonly used stem cell types, including mesenchymal SCs, embryonic SCs and induced pluripotent SCs [14–16]. Stem cell surface CXCR-4 binds with SDF-1α secreted by cancer cells, which stimulates stem cells to express more CXCR-4. Moreover, overexpression of the CXCR4 using gene transfection of human umbilical cord blood derived MSCs increased the migratory capacity of MSCs toward gliomas [15]. These results show the possibility to further increase migration capacity toward metastatic cancer *via* stem cell engineering. Other signaling pathways have been found, including urokinase type plasminogen activator (uPA) - uPA receptor (uPAR) and vascular endothelial growth factor receptor 2 (VEGFR2) [17, 18]. The degree of migration of stem cells towards a tumor is affected by diverse factors, including the nature of the stem cell, type of cancer and tumor microenvironment. Additional research is needed to better understand the factors influencing the migratory capacity of stem cells that allow the therapeutic potential for metastatic cancer treatment to be increased while reducing side effects of these stem cells.

### Strategies for metastatic cancer treatment using stem cells with anti-metastatic genes

**Figure 1:**
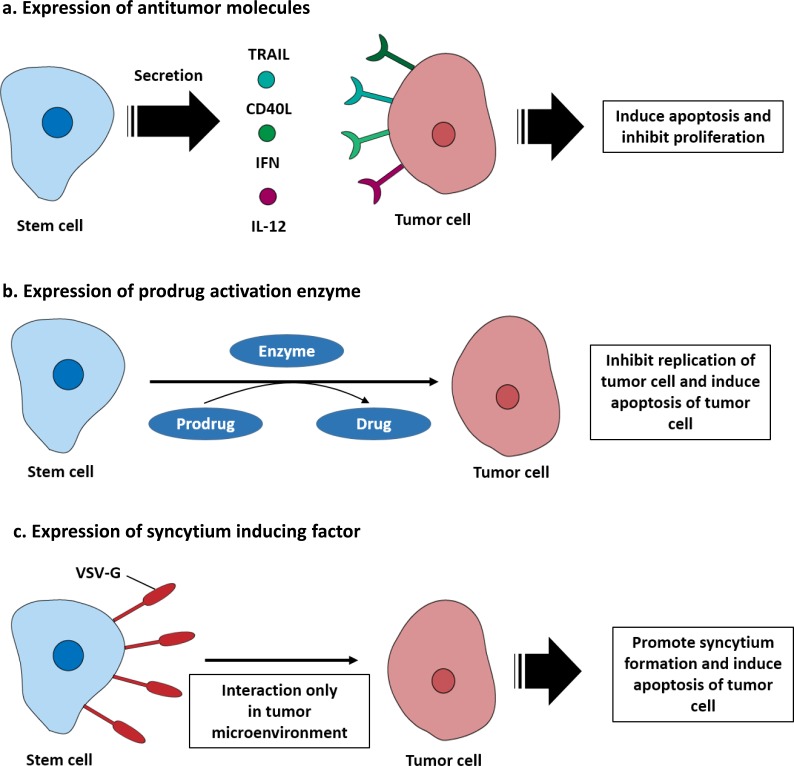
Engineered stem cells for metastatic cancer treatment **a.** Stem cells can be engineered to secrete antitumor molecules that function directly on tumor cells. For example, TRAIL, CD40L, IFN, and IL-12 bind to their receptors expressed by tumor cells and induce apoptosis. **b.** Stem cells can be engineered to express prodrug activating enzymes, including cytosine deaminase (CD) or carboxylesterase (CE), which converts a prodrug into a cytotoxic molecule. This induces suicide of the stem cell and apoptosis of tumor cells. **c.** Stem cells can be modified to express a syncytium formation factor on their membrane. The vesicular stomatitis virus glycoprotein (VSV-G) expressed on the stem cell surface bind with the tumor membrane. As a result, syncytium formation is promoted by stem cell in tumor microenvironment condition and induced tumor apoptosis.

Stem cells have intrinsic antitumor effects that occur through various factors secreted by stem cells and physical interactions of stem cells with tumor cells [19, 20]. However, unmodified stem cells are insufficient to treat cancers, and stem cells are typically engineered using viral transduction to express anticancer and anti-metastatic molecules. Stem cell secretion of therapeutic molecules can initially be divided into two categories depending on whether they directly target tumor cells or support immune system. Direct targeting molecules include the pro-apoptotic protein tumor necrosis factor related apoptosis inducing ligand (TRAIL), which binds to death receptor 4 (DR4) and DR5 and induces tumor cell apoptosis [21]. CD40 ligand is another pro-apoptotic molecule that binds to CD40 expressed on the tumor cell surface [22–24]. Membrane bound CD40 ligand triggered tumor cell apoptosis *via* activation of JNK/activation protein-1 and stimulated the secretion of both tumor necrosis factor alpha and interferon gamma, which ultimately activated the caspase 3/7 pathway [25, 26]. Neural stem cells derived from induced pluripotent stem cells transduced with baculovirus encoding CD40 ligand sufficiently inhibited tumor development in a preclinical model [27]. In addition, CD40 ligand expressing endothelial progenitor cells (EPCs) successfully migrated toward metastatic breast cancer lesions in the lung and induced tumor apoptosis [28]. Using cytokines such as the type I interferon family (IFN-α and β) to induce S-phase accumulation and apoptosis of tumor cells is another strategy for inhibition of proliferation pathways of the cancer and associated cells [29]. Interferon expressing stem cells have been shown to inhibit tumor growth in various preclinical cancer models [30, 31]. Secretion of interleukins that can stimulate immune system against tumor microenvironments has also been tested. Human MSCs have been engineered to secrete IL-12 and tested in preclinical metastatic hepatoma models. These studies revealed that the presence of IL-12 expressing stem cells could modify the immune profile of the tumor microenvironment. Moreover, the level of IFN-γ that is critical for innate and adaptive immunity activation increased. This change causes activation of natural killer cells and recruitment of tumor specific CD8+ T cells [32] as shown in Figure 1a. In addition, Table 1 summarizes the therapeutic gene transfer by stem cells for metastatic cancer treatment.

**Table 1:** Therapeutic gene transfer by stem cells for metastatic cancer treatment

Gene	Function	References
**TNF-related apoptosis inducing ligand (TRAIL)**	Binds to death receptor and induces tumor cell apoptosis	[18]
**CD40 ligand**	Stimulate the secretion of TNF-α and IFN-γ, which activate the caspase 3/7 pathway	[24,25]
**Type I interferon**	Induce S-phase accumulation and apoptosis of tumor cell	[27, 28]
**Interleukin 12 (IL-12)**	Stimulate the IFN-γ secretion and recruitment of tumor specific T-cell	[29]
**Cytosine deaminase**	Convert prodrug (5-FC) to activated drug (5-FU)	[32, 33]
**Carboxylesterase**	Convert prodrug (CPT-11) to activated drug (SN-38)	[34]
**Vesicular stomatitis virus glycoprotein (VSV-G)**	Induce cell to cell fusion and promote the formation of multinucleated syncytia, eventually causing cell death	[37]

### Strategies for metastatic cancer treatment using stem cells with prodrugs

Stem cell mediated suicide gene therapy is another strategy for killing tumor cells. Stem cells are engineered to express an enzyme that converts a non-toxic prodrug into a cytotoxic drug that can efficiently kill tumor cells *via* the bystander effect. Cytosine deaminase (CD) and 5-fluorocytosine (5-FC) are well-known suicide gene systems. *E. coli* cytosine deaminase can convert a prodrug, 5-FC, into its active drug, 5-FU. The metabolite of 5-FU (fluorodeoxyuridine monophosphate) binds to the nucleotide binding site of the thymidylate synthase and dNTP in tumor cells becomes imbalanced, which can cause DNA damage and cell apoptosis [33]. In addition, carboxylesterase converts the prodrug irinotecan (CPT-11) to the potent topoisomerase I inhibitor SN-38. Topoisomerase I catalyzes DNA unwinding, which is a critical step in DNA replication and transcription. SN-38 binds to the DNA-Topoisomerase I complex, inhibiting ligation of the nicked DNA strand. Moreover, the SN-38-DNA-Topoisomerase I complex interrupts the movement of DNA polymerase along the DNA strand and induces tumor cell apoptosis [34]. The CD-5-FC system has been used in modified MSCs and NSCs and applied in metastasized preclinical models, where it could selectively treat metastasized cancer and inhibit tumor growth [35, 36]. In addition, human NSCs expressing carboxylesterase have been shown to be effective in preclinical models of metastatic lung cancer [37]. Furthermore, stem cell mediated suicide gene therapy has the additional advantage of the stem cell being eliminated after its therapeutic effect, which reduces side effects owing to long term retention [38] (Figure 1b).

Other strategies for inducing antitumor effects have also been studied. For example, using the vesicular stomatitis virus glycoprotein (VSV-G), which is one of the fusogenic membrane glycoproteins (FMGs) in neural stem cells, is a notable strategy for targeting tumor microenvironments [39]. VSV-G expressed in the neural stem cell membrane can induce rapid and extensive cell-to-cell fusion and promote the formation of multinucleated syncytia with cancer cells, eventually causing cell death. To specifically kill tumor cells, they engineered a pH sensor of VSV-G and generated a novel VSV-G mutant that efficiently promotes syncytium formation at the tumor extracellular pH (pH 6.8), but not at pH 7.4. In a preclinical metastatic breast cancer model, this system successfully inhibited cancer progression following systemic stem cell administration [40] (Figure 1c).

### Limitations of the stem cells based cancer therapy

Cancer treatments using stem cells have made improvements in regards to specific targeting of tumors, but a few obstacles must still be overcome prior to clinical application. The main concern is tumorigenicity of the stem cell and cell fate after systemic administration. To prevent therapeutic stem cells from forming tumors or aberrantly differentiating in the host, the tumorigenicity or differentiation potential should be tested in preclinical models. In addition, immortalized stem cells with therapeutic gene inserts may solve the difficulty of mass culture of stem cells and enable their stability in tumors. Therefore, it is important to develop a new strategy for mass culture of stem cells to ensure the ability of a suicide gene to perfectly eliminate stem cells after therapy. Therapeutic modifications could adversely affect the safety of stem cells. Therapeutic stem cells or secreted proteins interrupt host tolerance to self-antigens, which provokes additional complications in the patient and immune responses that might impair therapy. Improvement of stem cell specificity through detailed investigations of tumor tropism could relieve possible side effects.

## ONCOLYTIC VIROTHERAPY

The idea of oncolytic virotherapy originates from clinical reports of cancer regression caused by viral infection and is currently being developed by genetically modifying viruses for the selective infection and destruction of cancer cells [41].

### Tumor specific targeting strategies

The specificity of oncolytic viruses for tumors is very important to clinical trials. Many viruses have a natural specific tissue tropism for cell surface proteins that are overexpressed by cancer cells. This characteristic is very useful for metastatic cancer tracing. For example, measles virus recognizes the surface receptor CD46 (complement regulatory protein) for cell infection. CD46 is a cofactor for inactivation of complement components that is often overexpressed in cancer cells [42]. Herpes simplex virus uses the herpesvirus entry mediator (HVEM) and nectins which are overexpressed on the surface of cancer cells [43]. Coxsackievirus can recognize cell surface glycoprotein (intracellular adhesion molecule 1; ICAM-1) and GPI-anchored membrane protein (decay accelerating factor; DAF), which are overexpressed in cancers including melanoma and breast cancer [44, 45]. The enterovirus family inhibits expression of CD155, a key ligand in NK cell-mediated suppression of metastases that is overexpressed by some cancer cells [46].

Oncolytic viruses can be engineered to directly bind to unique surface molecules of cancer cells. This modification could assign additional specificity for metastasized tumor cells by improving infection of tumor tissues and decreasing infection of healthy tissues. This specificity can be achieved by modifying or combination protein of virus that require for cancer cell recognition. For example, glioma cells overexpress CD16 and CD80/86, which bind with adenovirus serotype 3 [47, 48]. Based on these findings, a chimeric adenovirus vector (Ad5/3) contains the backbone of adenovirus serotype 5 fiber with an adenovirus, serotype 3 knob. The Ad 5/3 chimeric virus exhibits increased targeting capabilities for cell lines analyzed *in vitro* by at least ten-fold. These data suggest an improved safety of Ad 5/3 in the setting of malignant glioma [49]. The adenovirus Ad5/3-Δ24 was modified to bind to CD46, which are highly expressed in metastatic renal cancer and significantly increased antitumor effects in a preclinical model [50]. Other examples of engineered specificities include lentiviruses pseudotyped with Sindbis virus, which targeted human P-glycoprotein ectopically expressed on the surface of melanoma cells. This oncolytic virus successfully targeted metastatic melanoma cells after systemic administration by tail vein injection [51].

Another strategy is a tumor specific transcriptional targeting using tumor specific promoters and microRNA target sequences. This strategy can restrict virus replication in off-target tissues. For example, adenovirus replication is correlated with their ability to promote cell cycle entry into the G1 phase through the viral immediate early protein E1A. Therefore, tumor specificity is achieved by placing the E1A gene under transcriptional regulation of a tumor specific promoter [52], CXCR4 in breast cancer and prostate specific antigen (PSA) promoter in prostate cancer [53, 54]. Another strategy is the application of microRNA targeting oncolytic viruses based on the discovery of differential expression patterns of micro RNA in tumor and normal tissues [55]. For example, the expression of let-7 is functionally linked to tumors, regulating the over-expression of proto-oncogenes, and reflecting the differentiation state of tumors [56]. Incorporation of let-7 microRNA complementary sequences within the 3′ untranslated region (UTR) of the wild-type matrix protein of the VSV gene plays an essential role in viral replication and eliminates unwanted replication and associated toxicity in normal cells, but permits growth in cancer cells [57]. In adenovirus, insertion of liver specific microRNA (miR-122) binding sites in the 3′ UTR of the gene encoding E1A of an oncolytic adenovirus decreased its toxicity without sacrificing tumor killing activity in a model of pancreatic cancer metastasis to the liver [58].

### Strategies for metastatic cancer treatment using viruses

**Figure 2:**
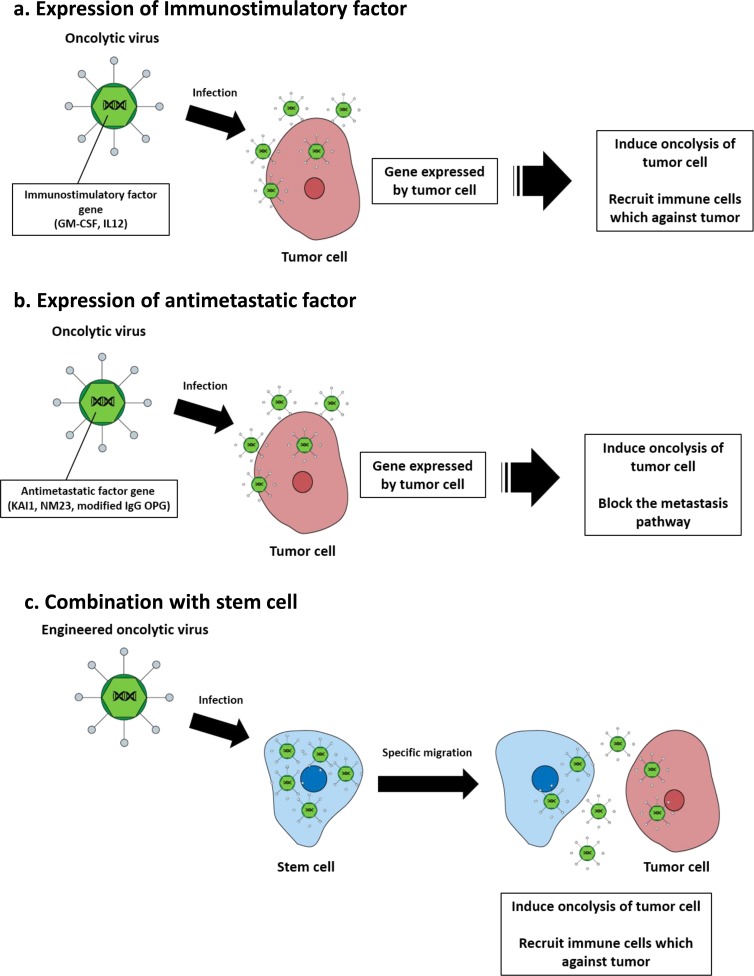
Oncolytic virotherapy strategies for metastatic cancer treatment **a.** Immunostimulatory factors (GM-CSF, IL-12) that are expressed in infected tumor cells recruit immune cells that induce tumor cell apoptosis. **b.** Anti-metastatic factors that are expressed in infected tumor cells block the metastasis pathway of the tumor cell and kill the tumor *via* their oncolytic capacity. **c.** Oncolytic virus infected stem cells replicate within the stem cells, which then migrate toward tumor lesions and release oncolytic viruses that infect tumor cells and induce oncolysis.

After oncolytic virus infection, cancer cells are destroyed by lysis. However, various strategies have been studied to achieve the maximum therapeutic efficacy for tumor cells. The therapeutic efficacy of oncolytic viruses can be enhanced using strategies that enable immunostimulatory factors to induce innate and adaptive immune responses against tumors. One successful strategy is the expression of granulocyte macrophage colony stimulating factor (GM-CSF), which stimulates stem cells to produce granulocytes and monocytes and stimulates adaptive immunity against tumor associated antigens [59]. T-VEC is the most advanced herpes simplex virus based oncolytic virus encoding the GM-CSF gene. In clinical studies, T-VEC was shown to offer superior benefits during treatment of metastatic melanoma [60]. A potent anticancer molecule, IL-12, is an interleukin produced by dendritic cells in immune response, and treatment of cancer stimulates the production of interferon-gamma (IFN-γ) and tumor necrosis factor-alpha (TNF-α) from T cells and NK cells [61]. In a preclinical model of metastatic pancreatic cancer, the IL-12 gene inserted adenovirus significantly inhibited tumor cell growth [62] (Figure 2a).

Transfer of metastatic suppressor genes or targeting metastasis related molecules is an effective strategy of targeting metastatic cancer. One of the tumor suppressor proteins, KAI1, plays a key role in downregulation of epidermal growth factor receptor (EGFR) signaling, which is associated with increased receptor desensitization and endocytosis [63]. An adenovirus expressing KAI1 is applied in an orthotopic mouse model with non-small cell lung cancer lymphatic metastasis, which has decreased lymphatic metastasis but not decreased primary tumor volume [64]. Reduced NM23 expression has been shown to be significantly associated with metastatic behavior in many cancer types [65–67]. Liver metastasis of ovarian cancer and animal survival time were measured after transfer of a recombinant adenovirus expressing NM23 into the preclinical model. A significant reduction in the number of animals developing liver metastases and prolongation of median survival time was observed relative to the untreated group [68]. Some studies have suggested that overexpression of the transforming growth factor beta (TGF-β) pathway is associated with breast cancer bone metastases [69–71]. Based on these results, modified adenoviruses expressing soluble form of transforming growth factor-beta receptor II (TGF-βRII) fused with human immunoglobulin Fc fragment could bind with TGF-β and successfully inhibit breast cancer with bone metastasis in a mouse model [72]. Similarly, an adenovirus expressing soluble osteoprotegerin (OPG) linked to the Fc portion of immunoglobulin G (IgG) is also effective at inhibiting the progression of bone metastasis of breast cancer [73]. Osteoprotegerin (OPG) acts as a decoy receptor for receptor activator of nuclear factor kappa-B ligand (RANKL), which allows sufficient microenvironmental conditions to influence cancer cell migration (Figure 2b) [74, 75].

Several studies have also explored the possibility of combining oncolytic viruses with stem cells to improve delivery [76]. The therapeutic efficacy of an oncolytic virus is determined by clearance of the virus by the host immune system following systemic and intratumoral administration. Stem cells infected with oncolytic virus migrated to the tumor and locally released undamaged oncolytic viruses. hMSCs have recently been shown to function as effective carriers to deliver oncolytic viruses. In a preclinical model, hMSC transduced with conditionally replicating adenoviruses significantly suppressed pulmonary metastasis of breast cancer through viral amplification in hMSCs [77]. In addition, stem cell delivery of oncolytic viruses has been shown to be effective in several preclinical cancer models, such as ovarian cancer and hepatocellular carcinoma [78, 79]. Thus, hMSCs may be an effective platform for the targeted delivery of oncolytic viruses to distant cancer sites such as metastatic breast cancer (Figure 2c). In addition, the therapeutic gene transfers by tumor-tropic viruses were shown for metastatic cancer treatment (Table 2).

**Table 2:** Therapeutic gene transfer by viruses for metastatic cancer treatment

Gene	Function	References
**Granulocyte macrophage colony-stimulating factor (GM-CSF)**	Stimulate adaptive immunity against tumor associated antigen	[56, 57]
**Interleukin – 12 (IL-12)**	Stimulate the IFN-γ secretion and recruitment of tumor specific T-cell	[58, 59]
**KAI1**	Inhibit the EGFR signaling, which associated with cell motility	[61]
**NM23**	Reduce the cell flexibility necessary for cytoskeleton plasticity and cell motility	[62–65]
**TGF-β receptor II (TGF-βRII)**	Bind with TGF-β and inhibit the TGF-β signaling pathway, which associated with cancer metastasis	[69]
**Osteoprotegerin linked Immunoglobulin G (OPG linked IgG)**	Bind with nuclear factor kappa-B ligand (RANKL), which associated with microenvironmental conditions to influence cancer cell metastasis	[70 - 72]

### Limitations of the virotherapy

The development of oncolytic viruses as therapeutic agents for metastatic cancer requires careful attention to establish appropriate clinical trial designs, as well as dosing regimens to minimize possible side effects. The most important technical challenge to overcome is the need to enhance tumor selectivity to decrease off target effects after systemic delivery of the oncolytic virus. The other major obstacle to successful application of viral therapy is neutralization of the virus by the host antibody. Many species of viruses are used in oncolytic virotherapy, and most people have already been exposed to the virus through previous vaccination or infection. Therefore, circulating antibodies can inhibit the oncolytic virus before it reaches the tumor site. Immune suppression agents and the aforementioned stem cell transport strategy is currently being investigated to solve this problem.

## CONCLUSIONS

Metastasis of cancer is one of the main factors leading to patient death. The unique properties of metastatic cancer, including their small size, high multiplicity and spread to multiple organs make it difficult to treat. Although conventional cancer treatment strategies have shown a lot of progress, they have been limited in metastatic cancer by recurrence of cancer, induction of drug resistance and systemic side effects after treatment. Accordingly, new strategies are needed to treat metastatic cancer.

Stem cell based and oncolytic virus strategies have many potential benefits. Stem cell based therapies are emerging as promising strategies to treat metastatic cancer. Multiple types of stem cells have been shown to exhibit natural tropism towards tumors. In addition, when engineered to express therapeutic agents including prodrug activation enzymes, cytokines and oncolytic viruses, these vehicles can deliver treatments to target sites of metastasized tumor lesions and effectively kill the cancer cell. Many metastatic cancer models have shown therapeutic stem cells to be safe and effective. In addition, clinical trials using promising therapeutic stem cells are under investigation and summarized in Table 3.

**Table 3:** Clinical trials for current stem cell cancer therapy

Stem cell	Name	Modification	Phase	References*
**Mesenchymal stem cell**	GX-051	IL-12 expression	1	NCT02079324
	N/A	Loading oncolytic adenovirus (ICOVIR-5)	1	NCT01864759
**Neural stem cell**	N/A	Cytosine deaminase expression	1	NCT02015819
	N/A	Carboxylesterase expression	1	NCT02192359

* NCT number is the identifier number on ClinicalTrials.gov.

Oncolytic virotherapy has rapidly advanced in a relatively short period through virological studies. In the early stage, oncolytic viruses destroyed tumors by their oncolysis ability alone; however, transduction of therapeutic transgenes and combination with other anti-tumor agents has enhanced the potency of the oncolytic virus platform. In addition, tumor selectivity has progressed *via* oncolytic virus particle modification, serotype changes and use of tumor specific activated promoters. Application of improved oncolytic viral constructs that can be delivered systemically or intratumorally will lead to effective treatments for metastatic cancer patients. Table 4 summarized promising clinical studies, which employ oncolytic viruses.

Despite these advances, additional research is needed to develop safer strategies and a lot of validation is required before preclinical models can be applied to humans. By understanding of metastatic processes and biological mechanisms that specifically drive each step of metastasis, we can develop more advanced therapeutic stem cells and strategies of oncolytic virotherapy, which are highly promising approaches to the treatment of metastatic cancer.

**Table 4:** Clinical trials for current oncolytic virotherapy

Virus	Name	Modification	Phase	References*
**Herpesvirus**	T-VEC	ICP34.5(-), GM-CSF expression	2	NCT02658812
	G207	ICP34.5(-), ICP6(-)	1/2	NCT00028158
**Adenovirus**	CG0070	E2F promoter, GM-CSF expression	3	NCT02365818
	ICOVIR-5	E2F promoter, E1A(-)	1	NCT01864759
	OBP-031	hTERT promoter	1/2	NCT02293850
	N/A	IL-12 expression	1	NCT00406939
**Retrovirus**	Toca511	Cytosine deaminase expression	1	NCT01470794
**Vaccinia virus**	JX-594	GM-CSF expression	1	NCT01380600
**Vesicular stomatitis virus**	VSV-hIFNβ	Interferon-β expression	1	NCT01628640

* NCT number is the identifier number on ClinicalTrials.gov.

## References

[1] Gupta GP, Massague J (2006). Cancer metastasis: building a framework. Cell.

[2] Weiss L. (2000). Metastasis of cancer: a conceptual history from antiquity to the 1990s. Cancer Metastasis Rev.

[3] Schroeder A, Heller DA, Winslow MM, Dahlman JE, Pratt GW, Langer R, Jacks T, Anderson DG (2012). Treating metastatic cancer with nanotechnology. Nat Rev Cancer.

[4] Engel J, Lategahn J, Rauh D (2016). Hope and Disappointment: Covalent Inhibitors to Overcome Drug Resistance in Non-Small Cell Lung Cancer. ACS Med Chem Lett.

[5] Jacot W, Pons E, Frenel JS, Guiu S, Levy C, Heudel PE, Bachelot T, D'Hondt V, Darlix A, Firmin N, Romieu G, Thezenas S, Dalenc F (2016). Efficacy and safety of trastuzumab emtansine (T-DM1) in patients with HER2-positive breast cancer with brain metastases. Breast Cancer Res Treat.

[6] Shojaei S, Gardaneh M, Rahimi Shamabadi A (2012). Target points in trastuzumab resistance. Int J Breast Cancer.

[7] Valabrega G, Montemurro F, Aglietta M (2007). Trastuzumab: mechanism of action, resistance and future perspectives in HER2-overexpressing breast cancer. Ann Oncol.

[8] McCubrey JA, Abrams SL, Fitzgerald TL, Cocco L, Martelli AM, Montalto G, Cervello M, Scalisi A, Candido S, Libra M, Steelman LS (2015). Roles of signaling pathways in drug resistance, cancer initiating cells and cancer progression and metastasis. Adv Biol Regul.

[9] Sosa MS, Bragado P, Aguirre-Ghiso JA (2014). Mechanisms of disseminated cancer cell dormancy: an awakening field. Nat Rev Cancer.

[10] Hanahan D, Weinberg RA (2000). The hallmarks of cancer. Cell.

[11] Klein CA (2003). The systemic progression of human cancer: a focus on the individual disseminated cancer cell—the unit of selection. Adv Cancer Res.

[12] Fidler IJ (2003). The pathogenesis of cancer metastasis: the ‘seed and soil’ hypothesis revisited. Nat Rev Cancer.

[13] Zhang S, Liu XZ, Liu ZL, Shang CZ, Hu QL (2009). Tropism mechanism of stem cells targeting injured brain tissues by stromal cell-derived factor-1. Chin J Traumatol.

[14] Koizumi S, Gu C, Amano S, Yamamoto S, Ihara H, Tokuyama T, Namba H (2011). Migration of mouse-induced pluripotent stem cells to glioma-conditioned medium is mediated by tumor-associated specific growth factors. Oncol Lett.

[15] Park SA, Ryu CH, Kim SM, Lim JY, Park SI, Jeong CH, Jun JA, Oh JH, Park SH, Oh W, Jeun SS (2011). CXCR4-transfected human umbilical cord blood-derived mesenchymal stem cells exhibit enhanced migratory capacity toward gliomas. Int J Oncol.

[16] Shi M, Li J, Liao L, Chen B, Li B, Chen L, Jia H, Zhao RC (2007). Regulation of CXCR4 expression in human mesenchymal stem cells by cytokine treatment: role in homing efficiency in NOD/SCID mice. Haematologica.

[17] Gutova M, Najbauer J, Frank RT, Kendall SE, Gevorgyan A, Metz MZ, Guevorkian M, Edmiston M, Zhao D, Glackin CA, Kim SU, Aboody KS (2008). Urokinase plasminogen activator and urokinase plasminogen activator receptor mediate human stem cell tropism to malignant solid tumors. Stem Cells.

[18] Schmidt NO, Przylecki W, Yang W, Ziu M, Teng Y, Kim SU, Black PM, Aboody KS, Carroll RS (2005). Brain tumor tropism of transplanted human neural stem cells is induced by vascular endothelial growth factor. Neoplasia.

[19] Qiao L, Xu Z, Zhao T, Zhao Z, Shi M, Zhao RC, Ye L, Zhang X (2008). Suppression of tumorigenesis by human mesenchymal stem cells in a hepatoma model. Cell Res.

[20] Schichor C, Albrecht V, Korte B, Buchner A, Riesenberg R, Mysliwietz J, Paron I, Motaln H, Turnsek TL, Jurchott K, Selbig J, Tonn JC (2012). Mesenchymal stem cells and glioma cells form a structural as well as a functional syncytium in vitro. Exp Neurol.

[21] Stuckey DW, Shah K (2013). TRAIL on trial: preclinical advances in cancer therapy. Trends Mol Med.

[22] Posner MR, Cavacini LA, Upton MP, Tillman KC, Gornstein ER, Norris CM (1999). Surface membrane-expressed CD40 is present on tumor cells from squamous cell cancer of the head and neck in vitro and in vivo and regulates cell growth in tumor cell lines. Clin Cancer Res.

[23] Alexandroff AB, Jackson AM, Paterson T, Haley JL, Ross JA, Longo DL, Murphy WJ, James K, Taub DD (2000). Role for CD40-CD40 ligand interactions in the immune response to solid tumours. Mol Immunol.

[24] Biagi E, Yvon E, Dotti G, Amrolia PJ, Takahashi S, Popat U, Marini F, Andreeff M, Brenner MK, Rousseau RF (2003). Bystander transfer of functional human CD40 ligand from gene-modified fibroblasts to B-chronic lymphocytic leukemia cells. Hum Gene Ther.

[25] Loskog A, Totterman TH (2007). CD40L - a multipotent molecule for tumor therapy. Endocr Metab Immune Disord Drug Targets.

[26] Ullenhag G, Loskog AS (2012). AdCD40L—crossing the valley of death?. Int Rev Immunol.

[27] Zhu D, Chen C, Purwanti YI, Du S, Lam DH, Wu C, Zeng J, Toh HC, Wang S (2014). Induced pluripotent stem cell-derived neural stem cells transduced with baculovirus encoding CD40 ligand for immunogene therapy in mouse models of breast cancer. Hum Gene Ther.

[28] Purwanti YI, Chen C, Lam DH, Wu C, Zeng J, Fan W, Wang S (2014). Antitumor effects of CD40 ligand-expressing endothelial progenitor cells derived from human induced pluripotent stem cells in a metastatic breast cancer model. Stem Cells Transl Med.

[29] Garrison JI, Berens ME, Shapiro JR, Treasurywala S, Floyd-Smith G (1996). Interferon-beta inhibits proliferation and progression through S phase of the cell cycle in five glioma cell lines. J Neurooncol.

[30] Ren C, Kumar S, Chanda D, Chen J, Mountz JD, Ponnazhagan S (2008). Therapeutic potential of mesenchymal stem cells producing interferon-alpha in a mouse melanoma lung metastasis model. Stem Cells.

[31] Ren C, Kumar S, Chanda D, Kallman L, Chen J, Mountz JD, Ponnazhagan S (2008). Cancer gene therapy using mesenchymal stem cells expressing interferon-beta in a mouse prostate cancer lung metastasis model. Gene Ther.

[32] Jeong KY, Lee EJ, Kim SJ, Yang SH, Sung YC, Seong J (2015). Irradiation-induced localization of IL-12-expressing mesenchymal stem cells to enhance the curative effect in murine metastatic hepatoma. Int J Cancer.

[33] Longley DB, Harkin DP, Johnston PG (2003). 5-fluorouracil: mechanisms of action and clinical strategies. Nat Rev Cancer.

[34] Kline CL, El-Deiry WS (2013). Personalizing colon cancer therapeutics: targeting old and new mechanisms of action. Pharmaceuticals (Basel).

[35] Yi BR, Kim SU, Choi KC (2016). Synergistic effect of therapeutic stem cells expressing cytosine deaminase and interferon-beta via apoptotic pathway in the metastatic mouse model of breast cancer. Oncotarget.

[36] Zhao D, Najbauer J, Annala AJ, Garcia E, Metz MZ, Gutova M, Polewski MD, Gilchrist M, Glackin CA, Kim SU, Aboody KS (2012). Human neural stem cell tropism to metastatic breast cancer. Stem Cells.

[37] Yi BR, Kim SU, Choi KC (2014). Co-treatment with therapeutic neural stem cells expressing carboxyl esterase and CPT-11 inhibit growth of primary and metastatic lung cancers in mice. Oncotarget.

[38] Stuckey DW, Shah K (2014). Stem cell-based therapies for cancer treatment: separating hope from hype. Nat Rev Cancer.

[39] Bateman A, Bullough F, Murphy S, Emiliusen L, Lavillette D, Cosset FL, Cattaneo R, Russell SJ, Vile RG (2000). Fusogenic membrane glycoproteins as a novel class of genes for the local and immune-mediated control of tumor growth. Cancer Res.

[40] Zhu D, Lam DH, Purwanti YI, Goh SL, Wu C, Zeng J, Fan W, Wang S (2013). Systemic delivery of fusogenic membrane glycoprotein-expressing neural stem cells to selectively kill tumor cells. Mol Ther.

[41] Cattaneo R, Miest T, Shashkova EV, Barry MA (2008). Reprogrammed viruses as cancer therapeutics: targeted, armed and shielded. Nat Rev Microbiol.

[42] Anderson BD, Nakamura T, Russell SJ, Peng KW (2004). High CD46 receptor density determines preferential killing of tumor cells by oncolytic measles virus. Cancer Res.

[43] Yu Z, Chan MK, P Oc, Eisenberg DP, Shah JP, Singh B, Fong Y, Wong RJ (2005). Enhanced nectin-1 expression and herpes oncolytic sensitivity in highly migratory and invasive carcinoma. Clin Cancer Res.

[44] Guo P, Huang J, Wang L, Jia D, Yang J, Dillon DA, Zurakowski D, Mao H, Moses MA, Auguste DT (2014). ICAM-1 as a molecular target for triple negative breast cancer. Proc Natl Acad Sci U S A.

[45] Shafren DR, Au GG, Nguyen T, Newcombe NG, Haley ES, Beagley L, Johansson ES, Hersey P, Barry RD (2004). Systemic therapy of malignant human melanoma tumors by a common cold-producing enterovirus, coxsackievirus a21. Clin Cancer Res.

[46] Carlsten M, Norell H, Bryceson YT, Poschke I, Schedvins K, Ljunggren HG, Kiessling R, Malmberg KJ (2009). Primary human tumor cells expressing CD155 impair tumor targeting by down-regulating DNAM-1 on NK cells. J Immunol.

[47] Ulasov IV, Rivera AA, Han Y, Curiel DT, Zhu ZB, Lesniak MS (2007). Targeting adenovirus to CD80 and CD86 receptors increases gene transfer efficiency to malignant glioma cells. J Neurosurg.

[48] Ulasov IV, Tyler MA, Zheng S, Han Y, Lesniak MS (2006). CD46 represents a target for adenoviral gene therapy of malignant glioma. Hum Gene Ther.

[49] Nandi S, Ulasov IV, Rolle CE, Han Y, Lesniak MS (2009). A chimeric adenovirus with an Ad 3 fiber knob modification augments glioma virotherapy. J Gene Med.

[50] Guse K, Ranki T, Ala-Opas M, Bono P, Sarkioja M, Rajecki M, Kanerva A, Hakkarainen T, Hemminki A (2007). Treatment of metastatic renal cancer with capsid-modified oncolytic adenoviruses. Mol Cancer Ther.

[51] Morizono K, Xie Y, Ringpis GE, Johnson M, Nassanian H, Lee B, Wu L, Chen IS (2005). Lentiviral vector retargeting to P-glycoprotein on metastatic melanoma through intravenous injection. Nat Med.

[52] Lu B, Makhija SK, Nettelbeck DM, Rivera AA, Wang M, Komarova S, Zhou F, Yamamoto M, Haisma HJ, Alvarez RD, Curiel DT, Zhu ZB (2005). Evaluation of tumor-specific promoter activities in melanoma. Gene Ther.

[53] Latham JP, Searle PF, Mautner V, James ND (2000). Prostate-specific antigen promoter/enhancer driven gene therapy for prostate cancer: construction and testing of a tissue-specific adenovirus vector. Cancer Res.

[54] Zhu ZB, Makhija SK, Lu B, Wang M, Kaliberova L, Liu B, Rivera AA, Nettelbeck DM, Mahasreshti PJ, Leath CA, Yamamoto M, Alvarez RD, Curiel DT (2004). Transcriptional targeting of adenoviral vector through the CXCR4 tumor-specific promoter. Gene Ther.

[55] Cawood R, Wong SL, Di Y, Baban DF, Seymour LW (2011). MicroRNA controlled adenovirus mediates anti-cancer efficacy without affecting endogenous microRNA activity. PLoS One.

[56] Mayr C, Hemann MT, Bartel DP (2007). Disrupting the pairing between let-7 and Hmga2 enhances oncogenic transformation. Science.

[57] Edge RE, Falls TJ, Brown CW, Lichty BD, Atkins H, Bell JC (2008). A let-7 MicroRNA-sensitive vesicular stomatitis virus demonstrates tumor-specific replication. Mol Ther.

[58] Suzuki T, Sakurai F, Nakamura S, Kouyama E, Kawabata K, Kondoh M, Yagi K, Mizuguchi H (2008). miR-122a-regulated expression of a suicide gene prevents hepatotoxicity without altering antitumor effects in suicide gene therapy. Mol Ther.

[59] Tong AW, Senzer N, Cerullo V, Templeton NS, Hemminki A, Nemunaitis J (2012). Oncolytic viruses for induction of anti-tumor immunity. Curr Pharm Biotechnol.

[60] Senzer NN, Kaufman HL, Amatruda T, Nemunaitis M, Reid T, Daniels G, Gonzalez R, Glaspy J, Whitman E, Harrington K, Goldsweig H, Marshall T, Love C, Coffin R, Nemunaitis JJ (2009). Phase II clinical trial of a granulocyte-macrophage colony-stimulating factor-encoding, second-generation oncolytic herpesvirus in patients with unresectable metastatic melanoma. J Clin Oncol.

[61] Kerkar SP, Leonardi AJ, van Panhuys N, Zhang L, Yu Z, Crompton JG, Pan JH, Palmer DC, Morgan RA, Rosenberg SA, Restifo NP (2013). Collapse of the tumor stroma is triggered by IL-12 induction of Fas. Mol Ther.

[62] Poutou J, Bunuales M, Gonzalez-Aparicio M, Garcia-Aragoncillo E, Quetglas JI, Casado R, Bravo-Perez C, Alzuguren P, Hernandez-Alcoceba R (2015). Safety and antitumor effect of oncolytic and helper-dependent adenoviruses expressing interleukin-12 variants in a hamster pancreatic cancer model. Gene Ther.

[63] Odintsova E, Sugiura T, Berditchevski F (2000). Attenuation of EGF receptor signaling by a metastasis suppressor, the tetraspanin CD82/KAI-1. Curr Biol.

[64] Takeda T, Hattori N, Tokuhara T, Nishimura Y, Yokoyama M, Miyake M (2007). Adenoviral transduction of MRP-1/CD9 and KAI1/CD82 inhibits lymph node metastasis in orthotopic lung cancer model. Cancer Res.

[65] Mandai M, Konishi I, Koshiyama M, Mori T, Arao S, Tashiro H, Okamura H, Nomura H, Hiai H, Fukumoto M (1994). Expression of metastasis-related nm23-H1 and nm23-H2 genes in ovarian carcinomas: correlation with clinicopathology, EGFR, c-erbB-2, and c-erbB-3 genes, and sex steroid receptor expression. Cancer Res.

[66] Muller W, Schneiders A, Hommel G, Gabbert HE (1998). Expression of nm23 in gastric carcinoma: association with tumor progression and poor prognosis. Cancer.

[67] Sirotkovic-Skerlev M, Krizanac S, Kapitanovic S, Husnjak K, Unusic J, Pavelic K (2005). Expression of c-myc, erbB-2, p53 and nm23-H1 gene product in benign and malignant breast lesions: coexpression and correlation with clinicopathologic parameters. Exp Mol Pathol.

[68] Li J, Zhou J, Chen G, Wang H, Wang S, Xing H, Gao Q, Lu Y, He Y, Ma D (2006). Inhibition of ovarian cancer metastasis by adeno-associated virus-mediated gene transfer of nm23H1 in an orthotopic implantation model. Cancer Gene Ther.

[69] Akhtari M, Mansuri J, Newman KA, Guise TM, Seth P (2008). Biology of breast cancer bone metastasis. Cancer Biol Ther.

[70] Kang Y, He W, Tulley S, Gupta GP, Serganova I, Chen CR, Manova-Todorova K, Blasberg R, Gerald WL, Massague J (2005). Breast cancer bone metastasis mediated by the Smad tumor suppressor pathway. Proc Natl Acad Sci U S A.

[71] Shipitsin M, Campbell LL, Argani P, Weremowicz S, Bloushtain-Qimron N, Yao J, Nikolskaya T, Serebryiskaya T, Beroukhim R, Hu M, Halushka MK, Sukumar S, Parker LM, Anderson KS, Harris LN, Garber JE (2007). Molecular definition of breast tumor heterogeneity. Cancer Cell.

[72] Zhang Z, Hu Z, Gupta J, Krimmel JD, Gerseny HM, Berg AF, Robbins JS, Du H, Prabhakar B, Seth P (2012). Intravenous administration of adenoviruses targeting transforming growth factor beta signaling inhibits established bone metastases in 4T1 mouse mammary tumor model in an immunocompetent syngeneic host. Cancer Gene Ther.

[73] Cody JJ, Rivera AA, Lyons GR, Yang SW, Wang M, Sarver DB, Wang D, Selander KS, Kuo HC, Meleth S, Feng X, Siegal GP, Douglas JT (2010). Arming a replicating adenovirus with osteoprotegerin reduces the tumor burden in a murine model of osteolytic bone metastases of breast cancer. Cancer Gene Ther.

[74] Castellano D, Sepulveda JM, Garcia-Escobar I, Rodriguez-Antolin A, Sundlov A, Cortes-Funes H (2011). The role of RANK-ligand inhibition in cancer: the story of denosumab. Oncologist.

[75] Koch L (2011). Cancer: RANKL inhibition-a new weapon against breast cancer?. Nat Rev Endocrinol.

[76] Kim J, Hall RR, Lesniak MS, Ahmed AU (2015). Stem Cell-Based Cell Carrier for Targeted Oncolytic Virotherapy: Translational Opportunity and Open Questions. Viruses.

[77] Stoff-Khalili MA, Rivera AA, Mathis JM, Banerjee NS, Moon AS, Hess A, Rocconi RP, Numnum TM, Everts M, Chow LT, Douglas JT, Siegal GP, Zhu ZB, Bender HG, Dall P, Stoff A (2007). Mesenchymal stem cells as a vehicle for targeted delivery of CRAds to lung metastases of breast carcinoma. Breast Cancer Res Treat.

[78] Mader EK, Butler G, Dowdy SC, Mariani A, Knutson KL, Federspiel MJ, Russell SJ, Galanis E, Dietz AB, Peng KW (2013). Optimizing patient derived mesenchymal stem cells as virus carriers for a phase I clinical trial in ovarian cancer. J Transl Med.

[79] Ong HT, Federspiel MJ, Guo CM, Ooi LL, Russell SJ, Peng KW, Hui KM (2013). Systemically delivered measles virus-infected mesenchymal stem cells can evade host immunity to inhibit liver cancer growth. J Hepatol.

